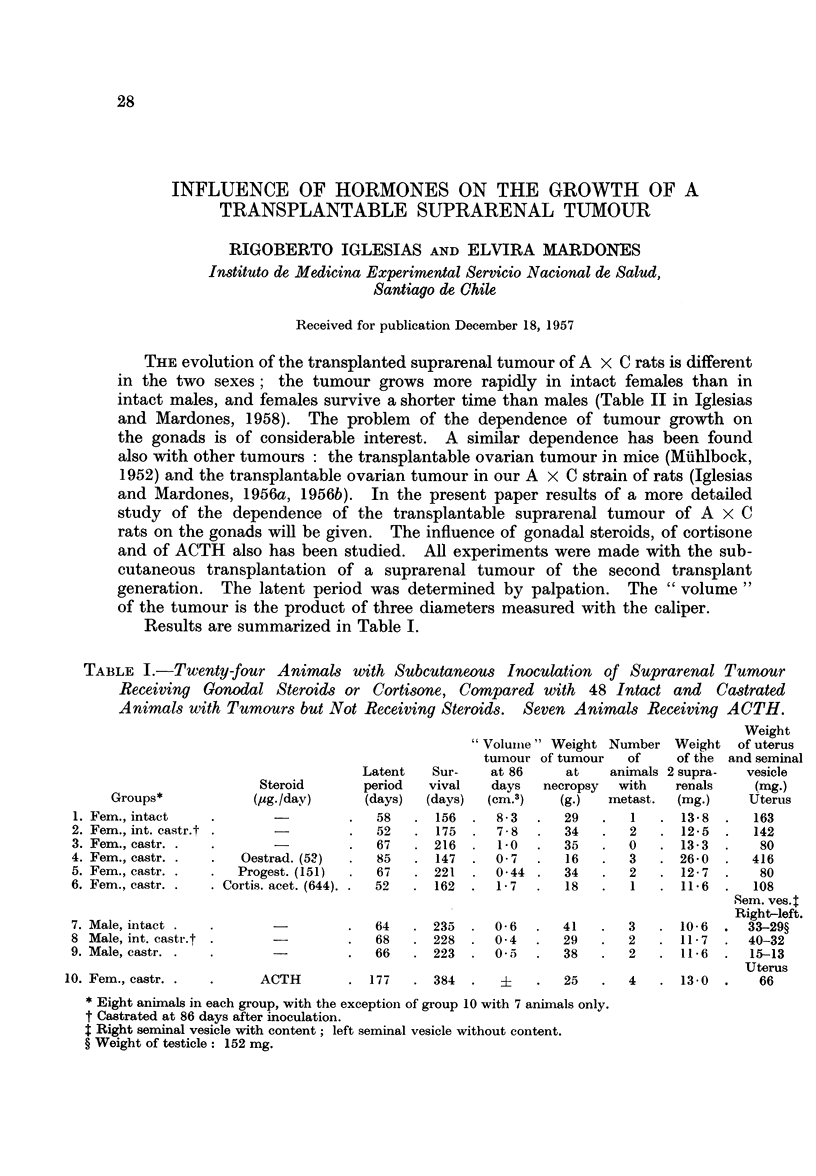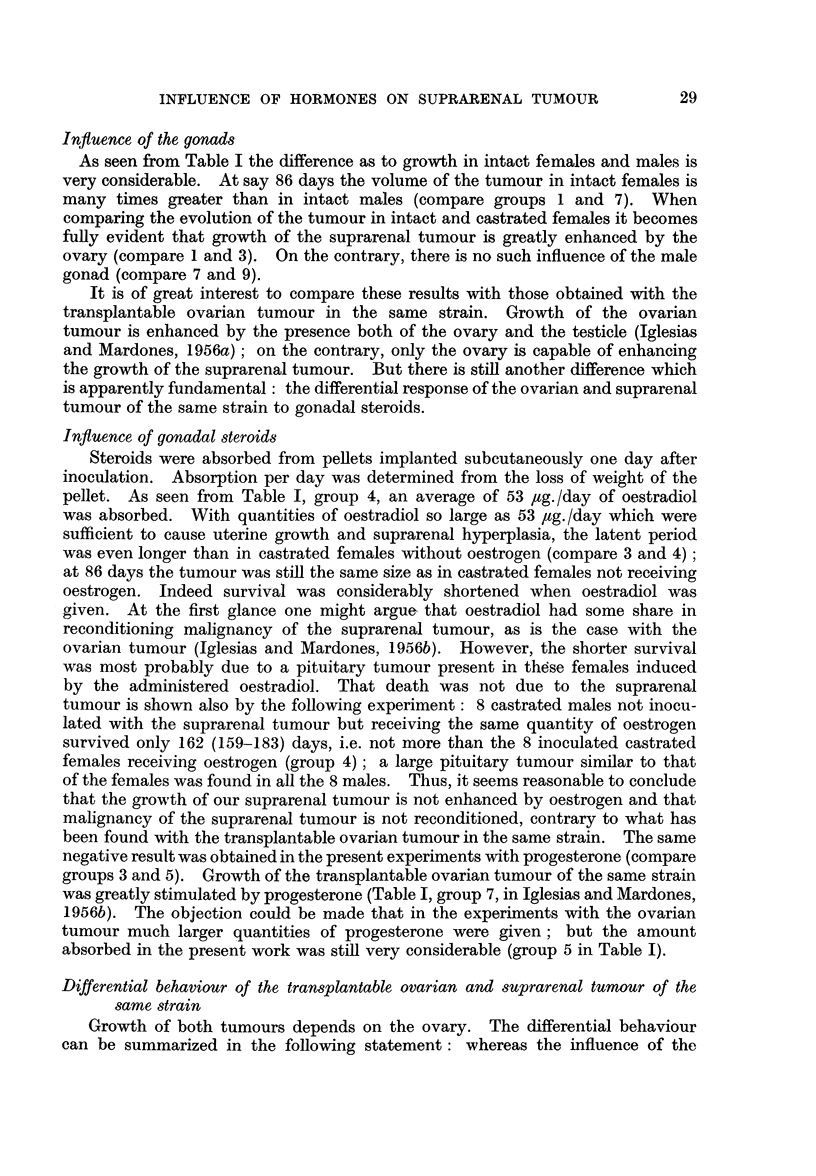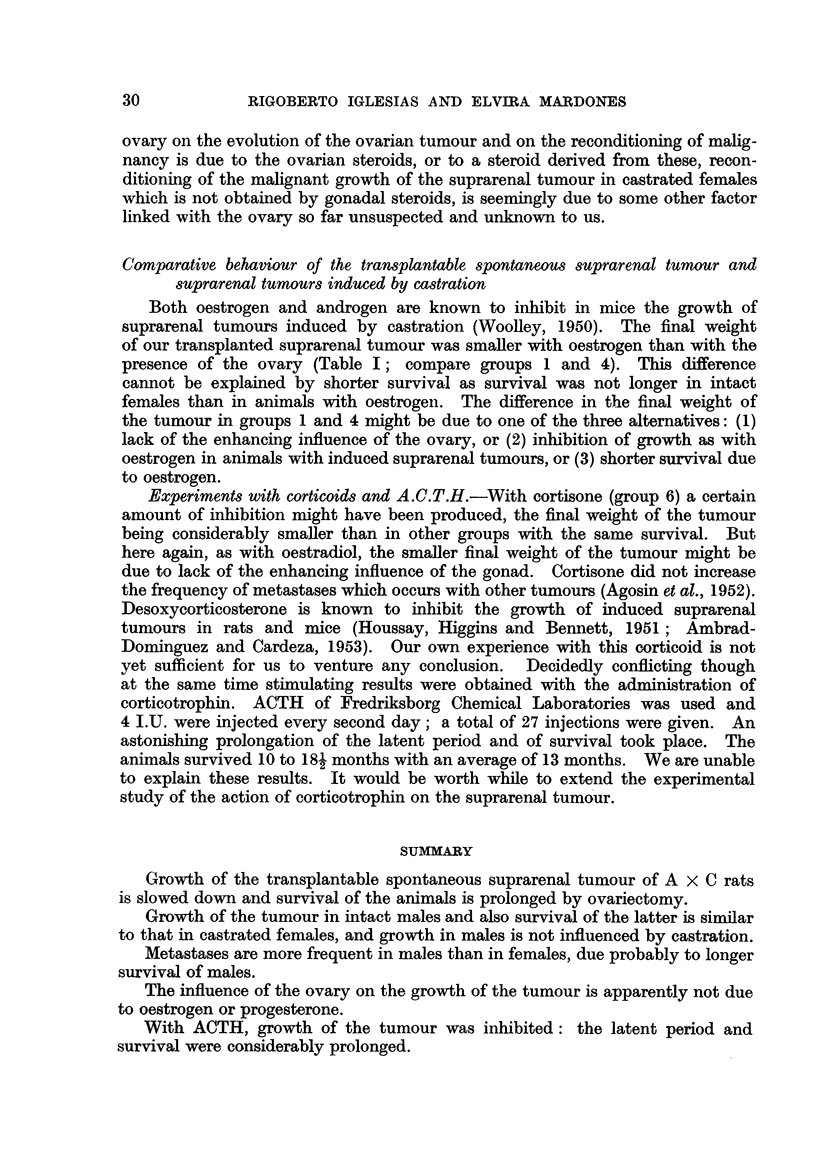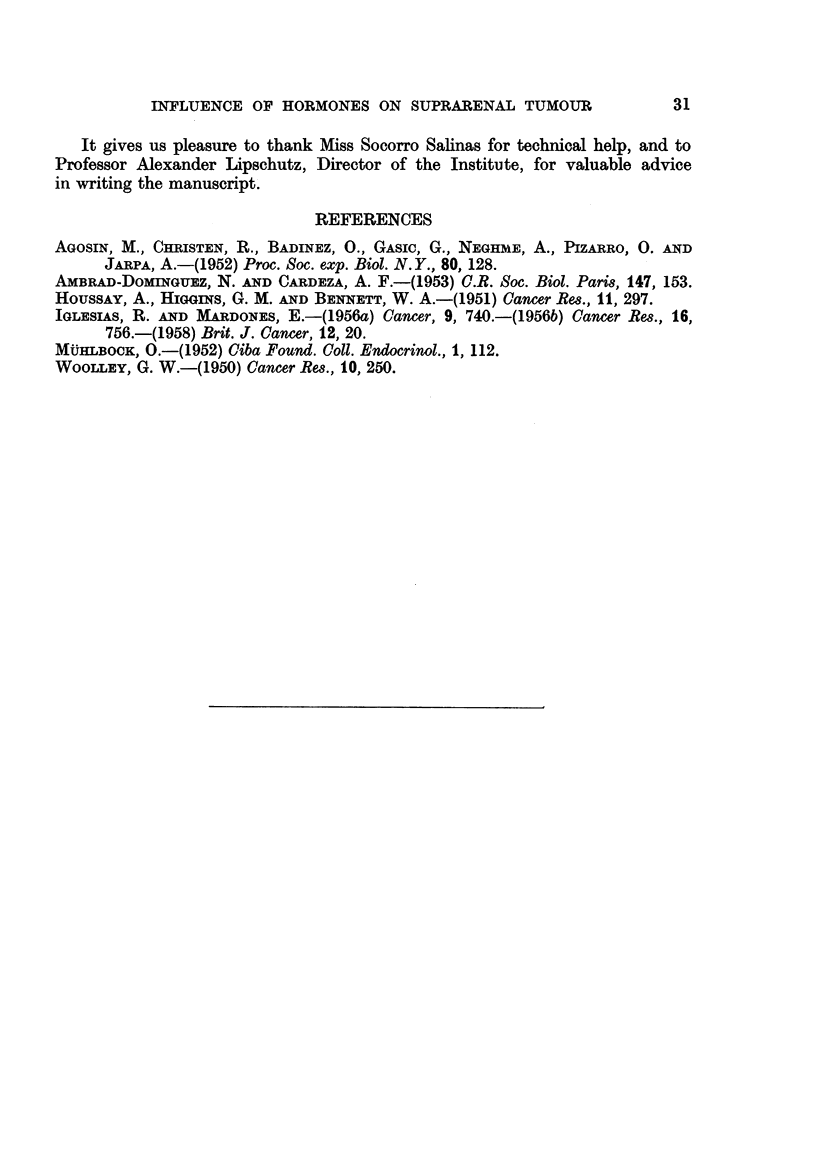# Influence of Hormones on the Growth of a Transplantable Suprarenal Tumour

**DOI:** 10.1038/bjc.1958.5

**Published:** 1958-03

**Authors:** Rigoberto Iglesias, Elvira Mardones


### INFLUENCE OF HORMONES ON THE GROWTH OF A TRANSPLANTABLE SUPRARENAL TUMOUR

RIGOBERTO IGLESIAS AND ELVIRA MARDONES

*Instituto de Medicina Experimental Servicio Nacional de Salud, Santiago de Chile*

Received for publication December 18, 1957

THE evolution of the transplanted suprarenal tumour of A × C rats is different in the two sexes; the tumour grows more rapidly in intact females than in intact males, and females survive a shorter time than males (Table II in Iglesias and Mardones, 1958). The problem of the dependence of tumour growth on the gonads is of considerable interest. A similar dependence has been found also with other tumours : the transplantable ovarian tumour in mice (Mühlbock, 1952) and the transplantable ovarian tumour in our A × C strain of rats (Iglesias and Mardones, 1956*a*, 1956*b*). In the present paper results of a more detailed study of the dependence of the transplantable suprarenal tumour of A × C rats on the gonads will be given. The influence of gonadal steroids, of cortisone and of ACTH also has been studied. All experiments were made with the subcutaneous transplantation of a suprarenal tumour of the second transplant generation. The latent period was determined by palpation. The " volume " of the tumour is the product of three diameters measured with the caliper.

Results are summarized in Table I.

TABLE I.—*Twenty-four Animals with Subcutaneous Inoculation of Suprarenal Tumour Receiving Gonodal Steroids or Cortisone, Compared with* 48 *Intact and Castrated Animals with Tumours but Not Receiving Steroids. Seven Animals Receiving ACTH.*

| Groups* | Steroid ($\mu$g./day) | Latent period (days) | Survival (days) | " Volume " tumour at 86 days (cm.$^3$) | Weight of tumour at necropsy (g.) | Number of animals with metast. | Weight of the 2 suprarenals (mg.) | Weight of uterus and seminal vesicle (mg.) Uterus |
|---|---|---|---|---|---|---|---|---|
| 1. Fem., intact | — | 58 | 156 | 8·3 | 29 | 1 | 13·8 | 163 |
| 2. Fem., int. castr.† | — | 52 | 175 | 7·8 | 34 | 2 | 12·5 | 142 |
| 3. Fem., castr. | — | 67 | 216 | 1·0 | 35 | 0 | 13·3 | 80 |
| 4. Fem., castr. | Oestrad. (53) | 85 | 147 | 0·7 | 16 | 3 | 26·0 | 416 |
| 5. Fem., castr. | Progest. (151) | 67 | 221 | 0·44 | 34 | 2 | 12·7 | 80 |
| 6. Fem., castr. | Cortis. acet. (644). | 52 | 162 | 1·7 | 18 | 1 | 11·6 | 108 |
| | | | | | | | | Sem. ves.‡ Right–left. |
| 7. Male, intact | — | 64 | 235 | 0·6 | 41 | 3 | 10·6 | 33–29§ |
| 8 Male, int. castr.† | — | 68 | 228 | 0·4 | 29 | 2 | 11·7 | 40–32 |
| 9. Male, castr. | — | 66 | 223 | 0·5 | 38 | 2 | 11·6 | 15–13 |
| | | | | | | | | Uterus |
| 10. Fem., castr. | ACTH | 177 | 384 | ± | 25 | 4 | 13·0 | 66 |

* Eight animals in each group, with the exception of group 10 with 7 animals only.
† Castrated at 86 days after inoculation.
‡ Right seminal vesicle with content ; left seminal vesicle without content.
§ Weight of testicle : 152 mg.

*Influence of the gonads*

As seen from Table I the difference as to growth in intact females and males is very considerable. At say 86 days the volume of the tumour in intact females is many times greater than in intact males (compare groups 1 and 7). When comparing the evolution of the tumour in intact and castrated females it becomes fully evident that growth of the suprarenal tumour is greatly enhanced by the ovary (compare 1 and 3). On the contrary, there is no such influence of the male gonad (compare 7 and 9).

It is of great interest to compare these results with those obtained with the transplantable ovarian tumour in the same strain. Growth of the ovarian tumour is enhanced by the presence both of the ovary and the testicle (Iglesias and Mardones, 1956*a*) ; on the contrary, only the ovary is capable of enhancing the growth of the suprarenal tumour. But there is still another difference which is apparently fundamental : the differential response of the ovarian and suprarenal tumour of the same strain to gonadal steroids.

*Influence of gonadal steroids*

Steroids were absorbed from pellets implanted subcutaneously one day after inoculation. Absorption per day was determined from the loss of weight of the pellet. As seen from Table I, group 4, an average of 53 $\mu$g./day of oestradiol was absorbed. With quantities of oestradiol so large as 53 $\mu$g./day which were sufficient to cause uterine growth and suprarenal hyperplasia, the latent period was even longer than in castrated females without oestrogen (compare 3 and 4) ; at 86 days the tumour was still the same size as in castrated females not receiving oestrogen. Indeed survival was considerably shortened when oestradiol was given. At the first glance one might argue that oestradiol had some share in reconditioning malignancy of the suprarenal tumour, as is the case with the ovarian tumour (Iglesias and Mardones, 1956*b*). However, the shorter survival was most probably due to a pituitary tumour present in these females induced by the administered oestradiol. That death was not due to the suprarenal tumour is shown also by the following experiment : 8 castrated males not inoculated with the suprarenal tumour but receiving the same quantity of oestrogen survived only 162 (159–183) days, i.e. not more than the 8 inoculated castrated females receiving oestrogen (group 4) ; a large pituitary tumour similar to that of the females was found in all the 8 males. Thus, it seems reasonable to conclude that the growth of our suprarenal tumour is not enhanced by oestrogen and that malignancy of the suprarenal tumour is not reconditioned, contrary to what has been found with the transplantable ovarian tumour in the same strain. The same negative result was obtained in the present experiments with progesterone (compare groups 3 and 5). Growth of the transplantable ovarian tumour of the same strain was greatly stimulated by progesterone (Table I, group 7, in Iglesias and Mardones, 1956*b*). The objection could be made that in the experiments with the ovarian tumour much larger quantities of progesterone were given ; but the amount absorbed in the present work was still very considerable (group 5 in Table I).

*Differential behaviour of the transplantable ovarian and suprarenal tumour of the same strain*

Growth of both tumours depends on the ovary. The differential behaviour can be summarized in the following statement : whereas the influence of the ovary on the evolution of the ovarian tumour and on the reconditioning of malignancy is due to the ovarian steroids, or to a steroid derived from these, reconditioning of the malignant growth of the suprarenal tumour in castrated females which is not obtained by gonadal steroids, is seemingly due to some other factor linked with the ovary so far unsuspected and unknown to us.

*Comparative behaviour of the transplantable spontaneous suprarenal tumour and suprarenal tumours induced by castration*

Both oestrogen and androgen are known to inhibit in mice the growth of suprarenal tumours induced by castration (Woolley, 1950). The final weight of our transplanted suprarenal tumour was smaller with oestrogen than with the presence of the ovary (Table I; compare groups 1 and 4). This difference cannot be explained by shorter survival as survival was not longer in intact females than in animals with oestrogen. The difference in the final weight of the tumour in groups 1 and 4 might be due to one of the three alternatives: (1) lack of the enhancing influence of the ovary, or (2) inhibition of growth as with oestrogen in animals with induced suprarenal tumours, or (3) shorter survival due to oestrogen.

*Experiments with corticoids and A.C.T.H.*—With cortisone (group 6) a certain amount of inhibition might have been produced, the final weight of the tumour being considerably smaller than in other groups with the same survival. But here again, as with oestradiol, the smaller final weight of the tumour might be due to lack of the enhancing influence of the gonad. Cortisone did not increase the frequency of metastases which occurs with other tumours (Agosin *et al.*, 1952). Desoxycorticosterone is known to inhibit the growth of induced suprarenal tumours in rats and mice (Houssay, Higgins and Bennett, 1951; Ambrad-Dominguez and Cardeza, 1953). Our own experience with this corticoid is not yet sufficient for us to venture any conclusion. Decidedly conflicting though at the same time stimulating results were obtained with the administration of corticotrophin. ACTH of Fredriksborg Chemical Laboratories was used and 4 I.U. were injected every second day; a total of 27 injections were given. An astonishing prolongation of the latent period and of survival took place. The animals survived 10 to 18½ months with an average of 13 months. We are unable to explain these results. It would be worth while to extend the experimental study of the action of corticotrophin on the suprarenal tumour.

### SUMMARY

Growth of the transplantable spontaneous suprarenal tumour of A × C rats is slowed down and survival of the animals is prolonged by ovariectomy.

Growth of the tumour in intact males and also survival of the latter is similar to that in castrated females, and growth in males is not influenced by castration.

Metastases are more frequent in males than in females, due probably to longer survival of males.

The influence of the ovary on the growth of the tumour is apparently not due to oestrogen or progesterone.

With ACTH, growth of the tumour was inhibited: the latent period and survival were considerably prolonged.

It gives us pleasure to thank Miss Socorro Salinas for technical help, and to Professor Alexander Lipschutz, Director of the Institute, for valuable advice in writing the manuscript.

### REFERENCES

AGOSIN, M., CHRISTEN, R., BADINEZ, O., GASIC, G., NEGHME, A., PIZARRO, O. AND JARPA, A.—(1952) *Proc. Soc. exp. Biol. N.Y.*, **80**, 128.

AMBRAD-DOMINGUEZ, N. AND CARDEZA, A. F.—(1953) *C.R. Soc. Biol. Paris*, **147**, 153.

HOUSSAY, A., HIGGINS, G. M. AND BENNETT, W. A.—(1951) *Cancer Res.*, **11**, 297.

IGLESIAS, R. AND MARDONES, E.—(1956*a*) *Cancer*, **9**, 740.—(1956*b*) *Cancer Res.*, **16**, 756.—(1958) *Brit. J. Cancer*, **12**, 20.

MÜHLBOCK, O.—(1952) *Ciba Found. Coll. Endocrinol.*, **1**, 112.

WOOLLEY, G. W.—(1950) *Cancer Res.*, **10**, 250.